# Long-term risk of chronic kidney disease and mortality in children after acute kidney injury: a systematic review

**DOI:** 10.1186/1471-2369-15-184

**Published:** 2014-11-21

**Authors:** Jason H Greenberg, Steven Coca, Chirag R Parikh

**Affiliations:** Department of Pediatrics, Section of Nephrology, Yale University School of Medicine, New Haven, CT USA; Department of Internal Medicine, Section of Nephrology, New Haven, CT and VA Medical Center, Yale University School of Medicine, West Haven, CT USA; Yale Program of Applied Translational Research, Yale University School of Medicine, 60 Temple Street, 6th Floor, Suite 6C, New Haven, 06510 CT USA

**Keywords:** Pediatrics, Acute kidney injury, Progression, Proteinuria, Hypertension, Chronic kidney disease, Long-term survival

## Abstract

**Background:**

Acute kidney injury (AKI) is associated with significant short-term morbidity and mortality in children. However, the risk for long-term outcomes after AKI is largely unknown.

**Methods:**

We performed a systematic review and meta-analysis to determine the cumulative incidence rate of proteinuria, hypertension, decline in glomerular filtration rate (GFR), and mortality after an episode of AKI. After screening 1934 published articles from 1985–2013, we included 10 cohort studies that reported long-term outcomes after AKI in children.

**Results:**

A total of 346 patients were included in these studies with a mean follow-up of 6.5 years (range 2–16) after AKI. The studies were of variable quality and had differing definitions of AKI with five studies only including patients who required dialysis during an AKI episode. There was a substantial discrepancy in the outcomes across these studies, most likely due to study size, disparate outcome definitions, and methodological differences. In addition, there was no non-AKI comparator group in any of the published studies. The cumulative incidence rates for proteinuria, hypertension, abnormal GFR (<90 ml/min/1.73 m^2^), GFR < 60 ml/min/1.73 m^2^, end stage renal disease, and mortality per 100 patient-years were 3.1 (95% CI 2.1-4.1), 1.4 (0.9-2.1), 6.3 (5.1-7.5), 0.8 (0.4 -1.4), 0.9 (0.6-1.4), and 3.7 (2.8-4.5) respectively.

**Conclusions:**

AKI appears to be associated with a high risk of long-term renal outcomes in children. These findings may have implications for care after an episode of AKI in children. Future prospective studies with appropriate non-AKI comparator groups will be required to confirm these results.

**Electronic supplementary material:**

The online version of this article (doi:10.1186/1471-2369-15-184) contains supplementary material, which is available to authorized users.

## Background

Pediatric acute kidney injury (AKI) is a significant health concern as its incidence has rapidly increased over the last 30 years [1, 2]. The increased incidence of AKI disproportionately affects children with chronic medical diseases who are hospitalized frequently and now living longer lives. This represents a shift in the epidemiology of AKI, in which AKI is now more often associated with complications of a child’s medical or surgical hospitalization compared to primary renal disease [3–7]. For hospitalized patients, AKI is independently associated with increased mortality and length of stay [8–11].

Until recently, AKI was considered a transient reversible syndrome. This paradigm has been refuted by a multitude of animal research and clinical studies. These studies support the hypothesis that an episode of AKI can cause permanent long-term kidney damage [12–14]. Animal studies have shown that AKI can lead to an irreversible reduction in peritubular capillaries causing hypoxia of renal parenchyma [15]. Additional studies show that AKI triggers molecular pathways that lead to prolonged cellular inflammation even after serum creatinine returns to baseline [16]. This inflammation contributes to fibrosis of the kidney and long-term renal dysfunction.

It is suggested that in humans as well, an episode of AKI causes renal inflammation and subsequent fibrosis that may result in chronic dysfunction [17, 18]. Depending on the severity of inflammation and the site of injury within the kidney, the phenotype of chronic dysfunction may be in the form of proteinuria, hypertension, chronic kidney disease (CKD), or end stage renal disease (ESRD). Multiple observational studies in adult populations have shown that AKI is an independent risk factor for CKD, ESRD, and mortality [19–25]. A systematic review and meta-analysis by Coca et al. determined that the long-term risk of CKD and ESRD in adult patients after AKI is higher compared with patients without AKI, with hazard ratios of 8.8 (95% CI 3.1-25.5) and 3.1 (95% CI 1.9-5.0) respectively [20]. While this adult research is striking, it is not sufficient to understand whether AKI is an independent risk factor for CKD in children. This is because CKD risk factors, such as diabetes, coronary artery disease, hypertension, and smoking are more prevalent in adults and influence the development of clinical outcomes after AKI. In a way, the pediatric population is an ideal group to study the actual contribution of AKI as they have fewer of these CKD risk factors affecting outcomes. Children also have a longer life expectancy with a longer time to manifest some of the possible outcomes after an episode of AKI.

It is important to understand the contribution of pediatric AKI to long-term renal and non-renal outcomes. This is imperative since there are no established outpatient management guidelines after resolution of an AKI episode. A recent editorial by Askenazi proposed that after an AKI children should have follow-up within 1 month of hospital discharge, quarterly for two visits, and then annually for two years [26]. If CKD or proteinuria is identified, patients would then be followed every 1 to 12 months as dictated by the KDIGO CKD guidelines [27]. This frequency of follow-up in CKD patients is dictated by GFR, albuminuria, underlying comorbid conditions, disease state, and risk of progression [27]. Proper follow-up of pediatric AKI patients could allow for earlier identification of renal outcomes and instituting therapy to limit disease progression. This follow-up will be critical as more children with chronic diseases are living longer and developing multiple AKI episodes over time [28, 29]. The goal of the present study was to conduct a systematic review of the published literature and estimate the risk for long-term sequelae of renal and non-renal outcomes after AKI.

## Methods

We searched the MEDLINE and EMBASE databases using the following terms: acute kidney injury (explode), AKI, ARF, chronic renal insufficiency (explode), chronic renal failure (explode), CKD, chronic disease, treatment outcome, follow-up studies, survival rate, quality of life, kidney injuries, and acute disease (refer to the on-line supplementary search strategy in Additional file 1 for a list of the search terms used). We also searched SCOPUS and the reference list of all reviewed articles to identify other eligible studies.

Studies were eligible for inclusion based upon the following criteria: 1) published from January 1985 onward; 2) mean outpatient follow-up after the episode of AKI was greater than 1 year; 3) pediatric cohort including patients aged 0–18 years at the time of AKI; 4) estimated GFR or measured GFR reported at a long-term follow-up visit. Data on proteinuria, hypertension, ESRD, or mortality as part of the follow-up data were also recorded. If a study had multiple time points when long-term follow-up data was obtained, the data from the last follow-up visit was used. There was no language restriction. Each article chosen by the primary reviewer was reviewed by a second reviewer to confirm eligibility.

Studies were excluded if they reported on fewer than 10 patients at the time of follow-up. We also excluded studies that exclusively focused on neonates or studies that had a majority of patients with primary renal disease (e.g. hemolytic-uremic syndrome, post-infectious acute glomerulonephritis).

### Data abstraction

Data was extracted by using a standardized data extraction form. The reviewers extracted data about characteristics of participants (number, age, and sex), clinical setting, type of study (prospective versus retrospective), dates of enrollment, definition of AKI, definition of CKD, and rate of the outcomes (proteinuria, CKD, hypertension, ESRD, mortality) in participants with AKI. Methodological quality was assessed using the Cochrane Collaboration risk of bias guidelines [30].

### Outcome measures and statistical analysis

Primary outcome measures were long-term cumulative incidence rate of proteinuria, hypertension, abnormal GFR (<90 ml/min/1.73 m^2^), GFR < 60 ml/min/1.73 m^2^, end stage renal disease, and mortality. Abnormal GFR was also used as an outcome in addition to GFR < 60 ml/min/1.73 m^2^ because many studies used abnormal GFR as a primary outcome without specifying the GFR of each patient.

For each clinical outcome the cumulative incidence rate per 100 patient-years of follow-up was calculated. To determine this rate we used the number of events as our numerator and the number of patient-years of follow-up as our denominator. If there was no outcome data on the patients who were not enrolled from the original hospitalized cohort, we excluded these patients in our patient-years calculation. We also reported the pooled incidence of each outcome which designates the frequency of each specific long-term outcome. Both cumulative incidence rate and pooled incidence are weighted values.

## Results

**Figure 1:**
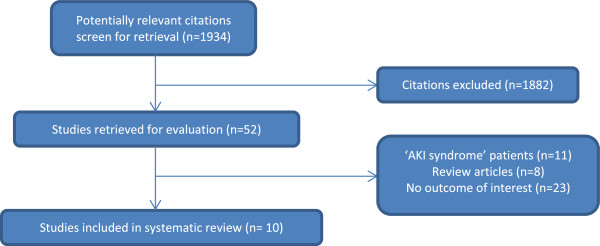
**Selection of studies.**

We identified 1934 citations from our MEDLINE and EMBASE online search and excluded 1882 citations based on the review of title and abstract. Full texts and reference lists for 52 articles were examined and 10 studies were included in the systematic review (Figure 1). The characteristics of the 10 studies included are described in Table 1. All of the primary studies reported on renal outcomes in children after discharge from a hospitalization during which they had an episode of AKI. All 10 studies were cohort studies and only one was conducted prospectively. Five of the studies enrolled hospitalized children with any stage of AKI and the other five only enrolled patients who required renal replacement therapy (RRT). Two studies reported on outcomes in AKI associated with cardiac surgery.

**Table 1:** **Characteristics of long-term follow-up studies of pediatric Acute Kidney Injury (AKI)**

Author, Year published	Country	Site	Study type	Setting	Severity	Episode of AKI	Age range of AKI	Mean F/U(y)	Mean age at F/U(y)
Askenazi [14], 2006	USA	SC	R	Hospitalized	Mixed AKI	1998 - 2001	6.4y^σ^	4	9.8
Buysse [50], 2008	Netherlands	SC	R	Meningitis	Mixed AKI	1988 - 2001	0.7-13.5y	10	15.9
Georgaki-Angelaki [45], 1989	U.K.	SC	R	Hospitalized	RRT	1971 - 1975	3d-10y	10	13
Mammen [41], 2012	Canada	SC	P	Hospitalized	Mixed AKI	2006 - 2008	0-18y	2^*^	3.0^*^
Mel [36], 2013	Israel	SC	R	Cardiac surgery	PD	1996 - 2004	0.12y^σ^	5^*^	8.4
Miler, 1997	Poland	SC	R	Hospitalized	Mixed AKI	1980 - 1990	3d-5y	6	-
Shaw [44], 1991	U.K.	SC	R	Cardiac surgery	RRT	1983 - 1988	2d - 2.5y	3	4.5
Slack [51], 2005	U.K.	MC	R	Meningitis	RRT	1996 - 1999	0.5-15y	4^*^	9.7
Viaud [46], 2011	France	SC	R	Hospitalized	RRT	1989 - 1996	1-14y	16^*^	21^*^
Wedekin [52], 2008	Germany	SC	R	Hospitalized Infant	Mixed AKI	1997 - 2003	0-1y	3^*^	-

Abbreviations: PD, peritoneal dialysis, RRT, renal replacement therapy, SC, single center, MC, multi-center, R, retrospective, P, prospective, d, days, y, years, F/U, follow-up, Mixed AKI, encompasses all types of AKI: mild AKI, severe AKI, and AKI requiring RRT, σ, no range available; only median age provided, *, median.

The 10 studies representing 8 different countries enrolled 346 patients. Six different definitions of AKI were used (Table 2). 60% (range 17-100%) of patients were enrolled from the surviving hospitalized cohort. Three of the primary studies excluded patients with pre-existing renal disease and the other 7 studies did not mention whether these patients were included or excluded. Long-term follow-up was also variable and was conducted from 2 years to 16 years after the episode of AKI, with a mean time of follow-up of 6.5 years. The outcome definitions and method of assessment for each primary studies is listed in Table 3.

**Table 2:** **Long-term outcome studies of pediatric Acute Kidney Injury (AKI)**

Author, Year published	AKI definition	Hospitalized cohort		Detailed follow-up cohort									
									Outcomes (%)				
		HospitalMortality, (%)	Acutely dialyzed during hospitalization (%)	Enrolled (%)	Mean follow-up (years)	Long-term mortality, (%)	# of Patients with follow-up	Abnormal GFR definition	Abnormal GFR	GFR <60	ESRD	Proteinuria	HTN
Askenazi [14], 2006	eGFR <75	71/245, (29)	21	17	4	35/174, (20)	29	GFR <90	14	7	9	28	21
Buysse [50], 2008	∆ SCr 100%	…	25	71	10	…	16	GFR <90	6	0	0	19	13
Georgaki-Angelaki [45], 1989	RRT	17/70, (24)	100	19	10	…	10	GFR <90	20	0	…	0	0
Mammen [41], 2012	AKIN criteria	40/425, (9)	17	33	2^*^	…	126	GFR ≤90	39	1	0	10	3
Mel [36], 2013	RRT	35/76, (46)	100	61	5^*^	15/41, (37)	25	GFR <90	4	0	0	0	0
Miler, 1997	AKI^Ω^	28/118, (24)	34	67	6	4/64, (6)	60	GFR <90	46	6	0	…	3
Shaw [44], 1991	RRT	23/34, (68)	100	100	3	0/11, (0)	11	abnormal mGFR	9	0	0	0	9
Slack [51], 2005	RRT	6/21, (29)	100	80	4^*^	…	12 ^β^	GFR <90	25	8	0	17	25
Viaud [46], 2011	RRT	16/52, (30)	100	36	16^*^	0/36, (0)	13	GFR <90	61	23	0	54	15
Wedekin [52], 2008	SCr >1.1	26/70, (37)	17	100	3^*^	7/44 (16)	44	abnormal SCr	0	…	…	…	…

The original hospitalized cohort is described along with the detailed follow-up cohort. The detailed follow-up cohort was assessed for long-term outcomes. Abbreviations: h, hour, RRT, renal replacement therapy, GFR, glomerular filtration rate (ml/min/1.73 m^2^), SCr, serum creatinine (in mg/dl. 1 mg, HTN, hypertension, UOP, urine output, AKIN criteria, Acute Kidney Injury Network classification system: ∆ SCr >0.3 mg/dL in 48 h, ∆ SCr ≥50%, or UOP <0.5 ml/kg/hr X 6 h, Ω, AKI diagnosed by “generally accepted criteria”, F/U, follow-up, …,not available, β, only patients with abnormal SCr at PICU discharge were followed, Enrolled (%) = # of patients with follow-up/# of patients who survived AKI admission, *, median.

**Table 3:** **Definition and method of attainment of study outcomes**

Author, Year published	Proteinuria definition	Proteinuria method	Hypertension definition	Hypertension method	Abnormal GFR definition	GFR estimation method
Askenazi [14], 2006	Urine alb/UCr ratio > 30 mg/g	NS	sys or dia >95% for age, sex, ht. HTN confirmed with 2 repeat visits	NS	GFR <90	Schwartz formula
Buysse [50], 2008	UPr/UCr >0.2 mg/mg	Avg of 3 first morning samples	sys > 95% for age, sex, ht	Avg of 3 BP measurements	GFR <90	Schwartz or Cockroft formula
Georgaki-Angelaki [45], 1989	NS	dipstick	abnormal BP for age	NS	GFR <90	Inulin Clearance
Mammen [41], 2012	Urine alb/UCr ratio ≥ 30 mg/g	Spot collection. If positive, first morning sample	sys or dia >95% for age, sex, ht. HTN confirmed with ABPM or 2 repeat visits	lowest of 3 BP readings	GFR ≤90	Schwartz formula or DTPA clearance
Mel [36], 2013	NS	Urinalysis	BP >90%	NS	GFR <90	Schwartz formula
Miler, 1997	NS	NS	NS	NS	GFR <90	Schwartz formula
Shaw [44], 1991	NS	dipstick	sys or dia >95% for age, sex, ht. HTN confirmed with 2 repeat visits	NS	Abnormal mGFR	DTPA clearance
Slack [51], 2005	>150 mg/day	24 hour collection	sys >95% for age, sex, ht	lowest of 3 BP readings	GFR <90	EDTA clearance
Viaud [46], 2011	UPr/UCr >0.5 mg/mg	NS	NS	NS	GFR <90	EDTA clearance
Wedekin [52], 2008	NP	NP	NP	NP	abnormal SCr	Schwartz formula

*Abbreviations:* *Alb* albumin, *UCr* urine creatinine, *UPr* urine protein, *NS* not specified, *Avg* average, *NP* not performed, *sys* systolic, *dia* diastolic, *HTN* hypertension, *ht* height, *BP* blood pressure, *DTPA* diethylenetriamine pentaacetate, *EDTA* ethylenediaminetetraacetic acid, *ABPM* ambulatory blood pressure monitoring, *SCr* serum creatinine.

A mean of 44% (range 17-100%) of the patients in the detailed follow-up cohort were acutely dialyzed for their AKI episode. 9 studies reported on the acute inpatient mortality of patients which averaged to 24% (range 9-68%) of patients dying with their acute illness.

### Proteinuria after AKI

The outcomes for each study are reviewed in Table 2. Eight studies reported on proteinuria. Multiple definitions of proteinuria were used in the primary studies (Table 3). Proteinuria was most commonly defined by a spot urine albumin to urine creatinine ratio greater than or equal to 30 mg/g or 2+ protein on urine dipstick. The rate of proteinuria was 3.1 per 100 person-years (95% CI 2.1-4.1) and the pooled incidence was 13.2% (95% CI 8.9-17.5%).

### Hypertension after AKI

Nine studies reported on hypertension. Hypertension was most commonly defined as repeated systolic or diastolic blood pressures greater than the 95^th^ percentile for age, gender, and height. The rate of hypertension after AKI was 1.4 per 100 person-years (CI 0.9-2.1) and the pooled incidence was 6.6% (CI 3.8-9.4%).

### Decline in GFR after AKI

All of the ten studies reported on abnormal GFR. In 8 of the 10 studies abnormal GFR was defined as a GFR < 90 ml/min/1.73 m^2^. Six different methods of estimating GFR were used, with 6 studies using the Schwartz formula (see Table 3) [31–35]. The rate of abnormal GFR was 6.3 per 100 person-years (CI 5.1-7.5) and the pooled incidence was 28.0% (CI 23.2-32.7%). Nine studies reported on the number of patients who developed CKD with a GFR < 60 ml/min/1.73 m^2^ after AKI, which was 0.8 per 100 person-years (CI 0.4-1.4) and the pooled incidence was 3.6% (CI 1.5-5.7%). Eight studies reported on ESRD at follow-up, which was 0.9 per 100 person-years (CI 0.6-1.4) and the pooled incidence was 0.4% (CI 0–0.9%). The pooled rate of impaired GFR and ESRD after AKI was 7.2 per 100 person-years.

### Long-term mortality after AKI

**Figure 2:**
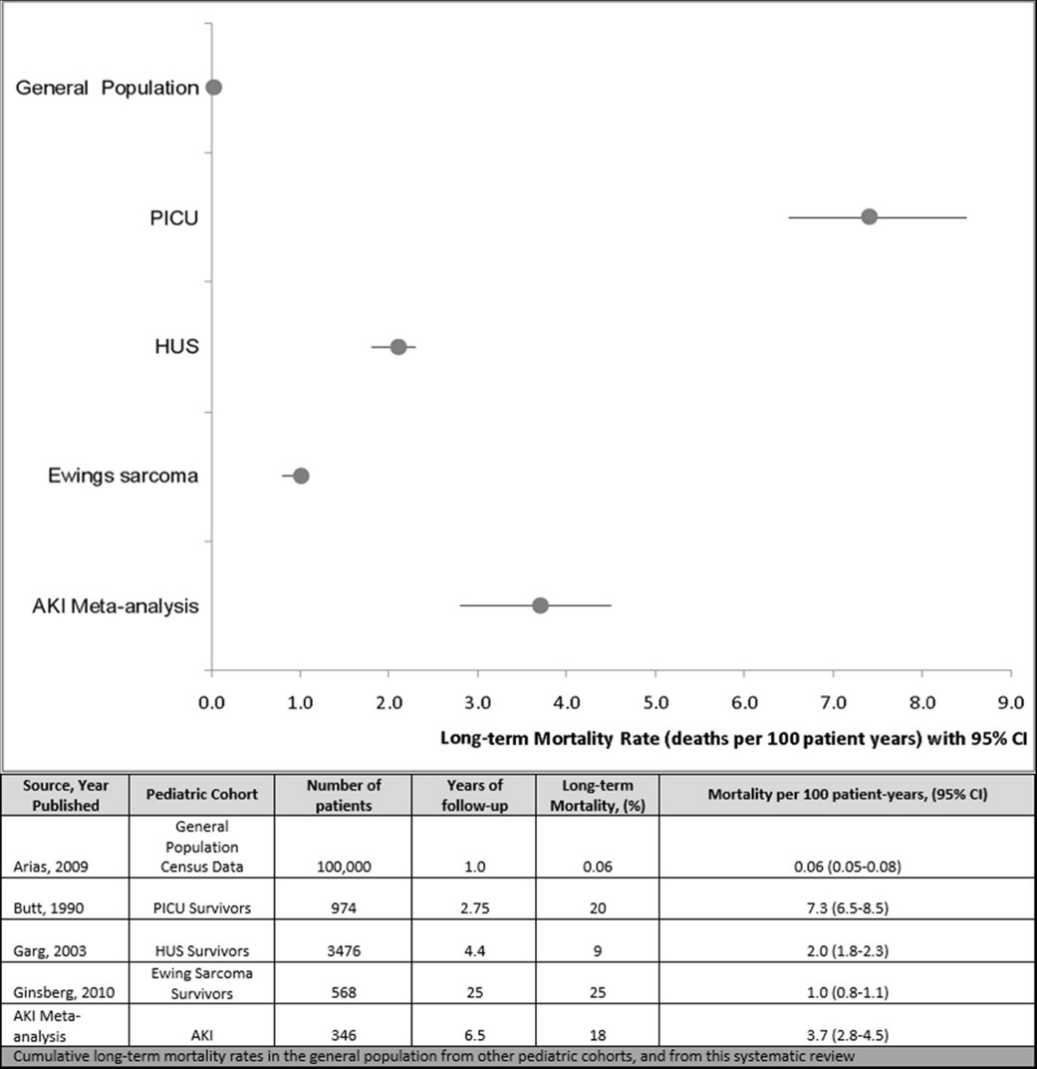
**Long-term mortality in various pediatric cohorts.**

**Figure 3:**
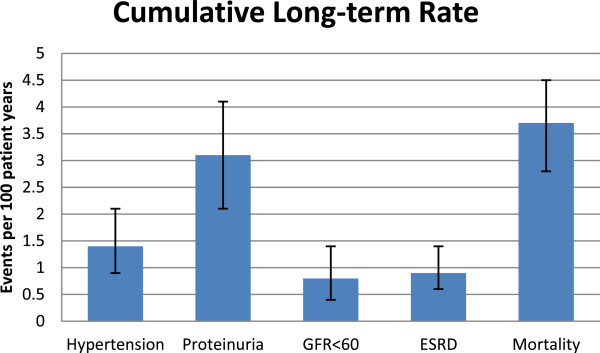
**Cumulative long-term rates of renal outcomes and mortality.**

Six studies reported on patients who had died after hospital discharge. The cumulative incidence rate of mortality was 3.7 per 100 person-years (CI 2.8-4.5) and the pooled incidence was 17.6% (CI 13.6-21.6%). As there was no comparator group within the studies, we compared long-term mortality rates after AKI with mortality rates of other pediatric cohorts detailed in Figure 2. We included a cohort of patients evaluated three years after discharge from the PICU, a meta-analysis of hemolytic uremic syndrome survivors evaluated after a mean follow-up of 4.4 years, survivors of Ewing sarcoma evaluated after 25 years, and the general pediatric population mortality rate (Figure 2) [14, 15, 36–39]. The AKI long-term mortality rate depicted in Figure 2 was comparable to the mortality rates found in other pediatric cohorts, but higher than the mortality rate of the general pediatric population [40]. Long-term event rates after AKI are displayed in Figure 3.

## Discussion

This is the first systematic review and meta-analysis evaluating the long-term outcomes after pediatric AKI. We calculated the cumulative incidence rates and pooled incidence of long-term kidney complications such as proteinuria, hypertension, abnormal GFR, CKD, and ESRD after an episode of AKI. None of the studies included a control group that would allow us to understand the attributable risk of these complications that would be related to AKI. The long-term risk of mortality after AKI was also high when compared with the general pediatric population. While some of the renal outcomes may be attributed to the fact that these children are sicker with many subsequent hospitalizations and comorbidities, it is likely that the episode of AKI itself is associated with permanent renal damage and progressive decline in kidney function.

As mentioned previously, experimental studies in animals describe several mechanisms by which AKI may cause long-term sequelae, as it induces hypoxia, inflammation, and fibrosis [12–16]. These studies suggest that AKI may play a causal role in subsequent renal outcomes. Multiple studies in adults have included control groups and demonstrate that AKI is an independent risk factor for renal outcomes [19–23]. The incidence rates of renal outcomes estimated in the adult literature are higher compared to our findings [20]. The systematic review by Coca et al. reported rates of CKD, ESRD, and mortality of 25.8 per 100 patient-years (range 3.4-72.2), 8.6 per 100 patient-years (range 0.63-28.1), and 16.8 per 100 patient-years (range 0.98-43.7) respectively [20]. This higher rate of outcomes in adults can be somewhat attributed to the greater prevalence of comorbid risk factors of ESRD such as CKD, diabetes, and heart failure in the adult population. At the index hospitalization approximately 1/3 of patients have CKD, 1/3 to ½ have diabetes, and 1/3 have heart failure in the adult AKI cohorts [19, 21–23, 25]. In addition, as we observed in pediatric cohorts, the etiology of AKI in adults is most commonly due to complications of a surgery or hospitalization, not primary kidney disease. Despite the tremendous growth of AKI clinical research over the past decade, clinical studies in children and adults have yet to establish that preventing an episode of AKI decreases the risk of development of CKD and premature mortality.

One of the long-term outcomes evaluated in the primary studies was a GFR between 60 and 90 ml/min/1.73 m^2^. In this review we estimated that this outcome was almost seven times more likely than a GFR <60 ml/min/1.73 m^2^. The clinical significance of having a GFR between 60 and 90 ml/min/1.73 m^2^ comes into question especially when it is estimated using the imprecise Schwartz formula. Use of the new bedside CKiD equation would likely have differing results as it has been shown to improve the accuracy of estimating GFR in children with CKD [31]. Even if an eGFR of 60–90 ml/min/1.73 m^2^ represents a true defect in glomerular filtration after the AKI, it is unclear whether this is a fixed abnormality of no clinical consequence or an abnormality that will progress to CKD and ESRD. The limited data from this review highlights this clinical uncertainty as no increased rates of abnormal GFR were found with increasing follow-up time. Moreover, it reinforces the need for research with a longer length of follow-up. A longer follow-up time will be beneficial, because even if it takes 25 years to manifest CKD after AKI, this would be clinically meaningful for a child who can have an additional 75 years of life expectancy after their AKI.

Two of the primary studies included hyperfiltration, or a GFR >150 ml/min/1.73 m^2^, as an outcome measure [14, 41]. We did not include hyperfiltration in our results as this clinical parameter is not used to identify patients at risk for CKD or diagnose CKD in clinical practice guidelines such as Kidney Disease Improving Global Outcomes (KDIGO) and Kidney Disease Outcomes Quality Initiative (KDOQI) [27, 42]. Hyperfiltration’s significance as a marker of kidney dysfunction and a predictor of GFR decline after pediatric AKI is unclear and has not been validated in a pediatric cohort [43].

The topic of long-term outcomes after pediatric AKI is attracting more focus from the medical community as children with chronic disease are living longer and possibly at risk for recurrent episodes of AKI. Although there has been no systematic review performed on this topic previously, a similar review and meta-analysis was performed on children after hemolytic uremic syndrome. Garg et al. studied long-term renal outcomes after Hemolytic Uremic Syndrome and estimated that the rate of GFR < 80 ml/min/1.73 m^2^ was 5.7 per 100 patient years, ESRD was 0.7 per 100 patient years, and mortality was 2.0 per 100 patient years [13]. In our systematic review the rate of abnormal GFR was 6.3, ESRD was 0.9, and mortality was 3.7 per 100 patient years.

While our review compels us to look closer at AKI as a risk factor for CKD in children, there were certain limitations to our results. First, 44% of patients included in these follow-up studies required renal replacement therapy during the index hospitalization for AKI. This is a higher percentage than is typically encountered in a cohort of pediatric AKI patients. More patients with severe AKI could have biased our results towards a higher cumulative incidence rate of renal dysfunction. There were also six distinct definitions of AKI used in the 10 different studies with some studies including only patients who required dialysis and others including any patient that had a 0.3 mg/dL increase in serum creatinine. The substantial difference between different AKI definitions could clearly affect the results in individual studies. Unfortunately a sensitivity analysis, where we stratified by AKI severity, was not feasible due to the small number of primary studies. Furthermore, many of the primary studies did not distinguish pre-renal causes of AKI from renal causes, including patients with any type of AKI and grouping them together. An AKI from volume depletion with a transient 0.3 mg/dl change in serum creatinine is less likely to have long-term sequelae as compared with an AKI from acute tubular necrosis. In order to definitively validate the long-term effects of AKI, standard definitions of AKI and CKD need to be uniformly adopted and used consistently in long-term studies [27, 42].

The primary studies included in this review have substantial differences of methodological quality with regards to selection of patients, loss to follow-up, and reporting of outcomes. We see that some studies report a high incidence of CKD after AKI and others reported none. One of the potential explanations for this variability is the differences in outcome definitions. The two studies by Shaw et al. and Georgaki-Angelaki et al. found no evidence of proteinuria using urine dipstick testing whereas studies by Askenazi et al. and Mammen et al. found a much higher incidence of proteinuria when using the more sensitive test for microalbuminiuria [14, 41, 44, 45]. On the other hand, Viaud et al. used a higher threshold test of a urine protein to creatinine ratio greater than 0.5 mg/mg to define proteinuria and found a 54% incidence [46]. This higher proportion of proteinuria may be attributed to the small sample size and a longer median follow-up time of 16 years. In addition, hypertension was diagnosed in some studies at one office visit whereas others required three separate visits for this diagnosis. Differing outcome definitions and methods of ascertainment highlight the importance of adhering to international consensus definitions when designing clinical studies. Due to this inter-study variability our pooled results should be interpreted carefully.

Other limitations of this study include small sample sizes and high rates of loss to follow-up. The latter may have introduced attrition bias, as the sicker population may be more likely to maintain contact with their physicians. These sicker patients may have inflated our calculated outcomes and led us to overestimate the amount of renal dysfunction after AKI.

With an increasing awareness of the long-term outcomes after AKI, more research needs to be performed. It remains to be determined whether all patients with AKI need to monitored or just those with more severe or prolonged AKI. Monitoring for renal outcomes would be relatively simple, likely involving a blood pressure check, a urine dip, and a serum creatinine. CKD-related complications such as proteinuria or hypertension could then be treated with a renin-angiotensin-aldosterone antagonist such as an ACE-inhibitor or an angiotensin receptor blocker. Primary care providers will likely become more involved with CKD screening as more children with chronic medical diseases are surviving their AKI and living longer.

## Conclusions

AKI appears to be associated with a higher risk of long-term renal and non-renal outcomes in children as compared with the general pediatric population. However, the variation in renal outcomes between primary studies was sufficiently large to advise caution before interpreting these findings. Additional research will be needed to more accurately calculate rates of long-term outcomes after AKI. The different outcome definitions used by primary studies underscore the importance of adhering to KDIGO AKI and CKD guidelines when defining clinical outcomes in future studies [27, 47]. Future research should include control groups where hospitalized patients without AKI are followed for renal sequelae. The lack of control groups in the primary studies clearly demonstrates a gap in the literature. Ongoing prospective long-term follow-up studies by the TRIBE-AKI (Translational Research Investigating Biomarker End-Points) consortium and the ASSESS-AKI Study (Assessment, Serial Evaluation, and Subsequent Sequelae of Acute Kidney Injury) was designed with control groups and should help to better define long-term outcomes after AKI in children [48–52]. In addition, there is a growing effort to study long-term AKI outcomes in neonates which was a focus of the NIH-sponsored Neonatal Acute Kidney Injury Workshop on April 9, 2013. A better understanding of pediatric AKI should provide opportunities for novel therapies and should direct the evolving guidelines of post-AKI outpatient management.

## Electronic supplementary material

Additional file 1: **Search Strategy.** (DOCX 14 KB)
