# Supplementary material for: Long-term risk of chronic kidney disease and mortality in children after acute kidney injury: a systematic review
Source: BMC Nephrol. 2014 Nov 21;15:184. doi: 10.1186/1471-2369-15-184 (PMC4251927; doi:10.1186/1471-2369-15-184)
Supplement: Supplementary file 1 — Additional file 1: Search Strategy. (DOCX 14 KB) [file 12882_2014_871_MOESM1_ESM.docx]

**Appendix 1: Search Strategy**

MEDLINE and EMBASE (January 1985 to February 2013) databases were searched using the following terms:

1. exp Acute Kidney Injury/

2. aki.mp.

3. acute kidney injury.mp.

4. acute renal failure.mp.

5. arf.mp.

6. 1 or 2 or 3 or 4 or 5

7. exp Renal Insufficiency, Chronic/

8. exp Kidney Failure, Chronic/

9. ckd.mp.

10. chronic kidney disease$.mp.

11. exp Chronic Disease/

12. 7 or 8 or 9 or 10 or 11

13. 6 and 12

14. limit 13 to "all child (0 to 18 years)"

15. exp Treatment Outcome/

16. exp Follow-Up Studies/

17. exp Survival Rate/

18. exp "Quality of Life"/

19. 6 and (15 or 16 or 17 or 18)

20. limit 19 to "all child (0 to 18 years)"

21. 20 not 14

22. exp Kidney/in [Injuries]

23. exp *Kidney/in

24. exp Acute Disease/

25. 23 and 24

26. limit 25 to "all child (0 to 18 years)"

27. 26 and (6 or 15 or 16 or 17 or 18)

28. limit 21 to yr="1990 -Current"

29. (followup or outcome or sequel$).mp.

30. 6 and 29

31. limit 30 to "all child (0 to 18 years)"

32. 31 not (14 or 28)
